# I’d rather have the girls eat first: a mixed-methods study on the nutritional health of migrant children in Chile

**DOI:** 10.3389/fnut.2026.1748412

**Published:** 2026-02-13

**Authors:** Teresita Rocha-Jimenez, Alejandra Carreño-Calderón, Janeth Solís de Ovando Calderón, Nicole Silva-Moreno, Marcela Oyarte, Maria Consuelo Robledo

**Affiliations:** 1 Society and Health Research Center, Escuela de Gobierno y Administración Pública, Universidad Mayor, Santiago, Chile; 2 SENTINET: Surveillance, Epidemiology, and New Technologies for Infectious Emerging Threats, Santiago, Chile; 3 Centro de Salud Global Intercultural, Facultad de Medicina Clínica Alemana, Facultad de Psicología, Universidad del Desarrollo, Santiago, Chile; 4 Centro de Interés Nacional para Investigación e Innovación en Niñez, Adolescencia, Resiliencia y Adversidad, IINARA, Santiago, Chile; 5 Millennium Institute for Care Research (MICARE), Santiago, Chile; 6 Doctoral Programme in Public Policy, Universidad Mayor, Santiago, Chile; 7 Doctoral Programme in Human and Social Sciences, Universidad de Almería, Almería, Chile; 8 Instituto de Salud Pública de Chile, Santiago, Chile

**Keywords:** acceptability, children, food insecurity, migration, Nutrition, South-America, undernutrition

## Abstract

**Introduction:**

Child malnutrition remains a persistent global challenge, disproportionately affecting migrant populations who experience barriers to health, food access, and social protection. In Chile, increasing migration from Latin America and the Caribbean (LAC) has reshaped the country’s nutritional landscape, revealing structural inequalities in child health. This study examines the nutritional status and food security of migrant children and adolescents in Chile, investigating how structural and cultural factors influence their access to adequate food and nutrition.

**Methods:**

A convergent mixed-methods design integrated quantitative analyses of national datasets, the National Socioeconomic Characterization Survey (CASEN 2022) and the Ministry of Health’s Monthly Statistical Records (REM 2019–2021) with 42 qualitative interviews conducted in 2023 with migrant caregivers and primary healthcare professionals across the regions of Tarapacá, O’Higgins, and Metropolitan Santiago. Quantitative analyses were conducted to describe nutritional status, food insecurity (using the ELCSA scale), and participation in school feeding programs (as measured by the JUNAEB). Qualitative data were thematically analyzed to capture the lived experiences of food access, healthcare, and cultural adaptation.

**Results:**

Quantitative findings revealed that migrant children exhibit lower rates of overweight and obesity, but a higher prevalence of undernutrition (2.9%) compared to Chilean peers (1.4%). Severe food insecurity affects 28.1% of migrant households, nearly double the rate among Chileans (15.9%). Qualitative findings highlight precarious living conditions, informal employment, and limited access to potable water and cooking facilities as barriers to adequate nutrition. Administrative restrictions linked to irregular migration status also hinder access to public food programs. Furthermore, cultural discrepancies between Chilean dietary guidelines and migrant food practices limits adherence and reinforces exclusion.

**Discussion:**

The study exposes a “double nutritional vulnerability” among migrant children, undernutrition arising from deprivation and potential overnutrition through dietary acculturation. These findings underscore the need for a dual-focus policy approach that ensures equitable access to nutrition and incorporates intercultural perspectives into child health programs. Expanding school feeding coverage regardless of migration status and culturally adapting nutritional interventions are essential steps toward reducing structural food insecurity and advancing child health equity in Chile.

## 1 Introduction

Child malnutrition is a global public health problem with consequences for children’s physical, psychological, and social development (1). In the context of migration, this challenge becomes even more complex, as migrant children often face social, economic, cultural, and access barriers to health and nutrition throughout the entire migration journey, making them particularly vulnerable (2). These conditions of social vulnerability make them more prone to food insecurity, malnutrition, and disruptions in eating practices (3). In addition, migrant children face greater disadvantages in health, psychosocial risk, exclusion from the school system, and multidimensional poverty compared to native children, especially when they live in unsafe or precarious conditions (4). In particular, the intersection between structural food insecurity, and sociocultural adaptation emerges as a complex issue that affects migrant childhood and has been little explored in the Chilean context (5–7). However, this reality is increasingly present in the countries of the Southern Cone and in their migratory flows, where structural conditions of inequality and social exclusion are intertwined with mobility processes and deepen the gaps in child health and nutrition (2). Thus, understanding these interrelated dynamics is essential for addressing the structural and cultural determinants in Chile that influence migrant children’s nutritional well-being and for guiding actions that uphold their right to food and health.

Child nutritional health mirrors broader social inequalities, which often restrict children’s access to adequate food—both in terms of quality, quantity, and regularity, thereby contributing to what is known as food and nutritional insecurity (FNI) (8). As highlighted by the Food and Agriculture Organization (9), this condition disproportionately affects children, women, and other vulnerable groups, with migrant children being particularly impacted (3, 10).

FNI manifests in various ways, ranging from overt hunger to nutrient-poor diets and, paradoxically, even overweight and obesity, conditions that can coexist within the same community or household (3). To understand FNI, it is essential first to consider its counterpart—food and nutritional security (FNS)—which is defined as the sustainable guarantee of access to sufficient, safe, nutritious, and culturally appropriate food (9). FNS is a multidimensional concept that extends beyond the mere absence of hunger or the availability of food; it encompasses stability, quality, and cultural adequacy of diet—dimensions that are particularly relevant in migratory contexts. Migration itself constitutes a social determinant of health (11), influencing each of these dimensions. Ensuring FNS, therefore, requires guaranteeing regular access to adequate and nutritious food while respecting the cultural identities and food practices of diverse groups (7, 12). However, constrained access to adequate food and nutrition often characterizes the entire migration journey, since the structural conditions that weaken FNS are frequently present even before migration.

In many countries of origin, children already face FNI, one of the main factors driving families to migrate to other Latin American countries, where limited food variety and insufficient availability continue to pose challenges (13–16). For example, a study of Venezuelan migrant families in Colombia revealed that widespread food and nutritional insecurity in their home country was a key driver of migration, as many families faced severe difficulties in obtaining sufficient and nutritious food (10). Participants described how the combined effects of economic collapse, political instability, and frequent shortages left them unable to feed their children adequately. Migration thus emerged as a survival strategy to restore access to food. In Guatemala, one of the countries with the poorest progress in reducing child malnutrition in the region, research indicates that international migration, particularly by fathers, can function as a strategy to alleviate children’s FNI through the income provided by remittances. However, the same study found that in households where fathers had migrated within the previous year, children under three years of age faced a higher risk of growth delays. Although remittances constitute a vital source of household income, they do not fully offset the negative consequences of prolonged parental absence during early childhood, a critical period for growth and development (17).

Beyond these dynamics at the origin, the transit stage of migration further exposes children to heightened nutritional risks. In the Peruvian context, Vargas-Machuca et al. (18) conducted a study at the Binational Border Healthcare Center in Tumbes to assess the nutritional status of Venezuelan children under five years of age arriving in the country. The findings revealed that approximately 3% of these children experienced acute malnutrition, while chronic malnutrition affected up to 18%, and anemia was present in one-quarter to one-third of the sample. During the migratory journey, less than one-fifth of children met the minimum recommended meal frequency, and many did not receive adequate solid or semi-solid foods due to the logistical and financial constraints inherent to migration (18).

In host countries, Pico et al. (10) note that while families may experience greater access to food at more affordable prices, serious challenges remain, such as labor precarity, informality, and low household income, which continue to limit access to truly nutritious food. This vulnerability is further exacerbated by language barriers, precarious housing, and the lack of institutional support networks, which hinder integration and the realization of the right to food in host context (19).

In the host country context, a study of migrant and Chilean schoolchildren in Antofagasta during the COVID-19 pandemic found that 73.3% experienced some degree of food and nutritional insecurity (FNI), with similar overall prevalence among nationals and migrants; however, severe FNI was more frequent among migrant students (20). Interestingly, while food security among Chilean families declined as household size increased, the opposite was observed among migrant families, suggesting that family cooperation and support networks play a protective role. This finding is consistent with Vilar-Compte et al. (21), who identified an inverse relationship between FNI and social support, as resource sharing and informal exchange mechanisms within migrant communities may help sustain food availability (21).

Malnutrition includes both undernutrition, which appears as deficiencies in essential nutrients reflected in low weight, stunting, or wasting, and overnutrition, expressed through overweight and obesity (1). The coexistence of these contrasting forms is known as the double burden of malnutrition, which is particularly prevalent in contexts characterized by social inequality and migration (8, 22, 23). This situation reflects the tension between persistent food and nutritional insecurity and the growing exposure to calorie-dense, ultra-processed diets, shaped by precarious living conditions and unequal access to healthy foods (6, 21, 23).

Research from high-income countries shows that migrant children often experience both forms of malnutrition. A systematic review conducted on malnutrition among migrants and refugees found that rates of overweight and obesity range from 11 to 42%, while stunting and underweight vary between 0.3 and 17% (24). Similar findings have been reported among Hispanic children in the United States, who display higher obesity rates together with deficiencies in micronutrients such as vitamin D, iron, folate, and iodine (23). In Mexico, children in households connected to migration or remittances also show an increased risk of overweight and obesity (21). These outcomes are influenced by acculturation processes, which refer to the cultural changes that occur through continuous contact with a new society (25), as well as the expansion of the global food industry (23). Migration alters eating behaviors, as food is a cultural practice deeply embedded in family life and identity (26–28). Adaptation to dominant food cultures can lead to increased consumption of industrialized products and less healthy diets, particularly when migrants transition from low-income to high-income countries (19).

Studies with Hispanic populations in the United States show that lower levels of acculturation are associated with more traditional diets rich in fruits, rice, and beans, whereas higher acculturation corresponds to greater consumption of fast food, sugar, and soft drinks (29, 30). Acculturation also influences family dynamics. Mothers who adopt assimilative parenting strategies tend to develop less healthy eating patterns, while higher parental education is associated with better diet quality (31, 32). In low- and middle-income countries, these dynamics are particularly significant because child undernutrition continues to coexist with rising rates of overweight and obesity (22). Additionally, a comparative study on health and lifestyle among migrant and native children in Chile found that, overall, migrant children showed better physical and nutritional indicators than Chilean children (32). Like other developing countries, Chile has undergone a notable epidemiological transition marked by a decline in infant mortality and malnutrition, among other indicators (33). An important part of this transition is marked by an improvement in the country’s socioeconomic levels, greater excess nutrients and a decrease in physical activity among children. Among the public policies that have guaranteed access to food for children, the establishment of the National School Assistance and Scholarship Board (Junta Nacional de Auxilio Escolar y Becas JUNAEB) stands out. Since 1964, it has provided free meals to all children attending public educational institutions (34, 35). These food programmes have had a significant impact not only on the eradication of child malnutrition, but also on the fight against poverty and the increase in school enrolment in various countries (36). Given this context, and considering the intersection between structural food insecurity, and sociocultural adaptation as a complex issue that affects migrant childhood and remains underexplored in the Chilean context, the aim of this study is to analyze the nutritional status of migrant children and adolescents, identifying both the barriers and enabling factors that influence the effective exercise of their right to adequate access to food and nutritional health through a mixed-methods study.

A mixed-methods design was selected because the nutritional status of migrant children and adolescents is shaped by both quantifiable factors and complex social determinants that cannot be fully understood through a single methodological lens. Quantitative data allow us to assess and compare nutritional outcomes between migrant and non-migrant children and adolescents, providing measurable evidence of inequities in access to food and nutritional health. However, to meaningfully interpret these disparities, it is essential to explore the contextual realities that underlie them. The qualitative component complements and deepens the quantitative findings by capturing caregivers’ lived experiences, including the structural barriers, enabling conditions, and sociocultural influences that affect their ability to exercise their right to adequate nutrition. This convergent integrated approach therefore strengthens the validity and policy relevance of the findings, offering a comprehensive understanding of the coexistence of multiple forms of malnutrition among children and adolescents in Chile, and illustrating disparities in nutrition realities.

## 2 Materials and methods

### 2.1 Study design

The study employed a convergent mixed-methods design, combining a quantitative descriptive component and a qualitative exploratory phase (37). The quantitative component used data bases collected from 2019–2021 (REM) and another in 2022 (CASEN). The qualitative component includes data collected in 2023. Both components were analyzed concurrently, as each addressed distinct aspects of the research objective. Triangulation was employed to ensure the validity and comprehensiveness of the findings (37). The quantitative component examined the social and nutritional dimensions of migrant childhood in Chile using population-based surveys and administrative records. The qualitative phase explored the experiences of Latin American and Caribbean (LAC) migrant adults living in Chile, as well as healthcare providers—including migrant caregivers of adolescents and primary healthcare professionals—regarding access to primary healthcare services related to the nutritional health of migrant children.

#### 2.1.1 Study setting

This study was conducted in the regions of Tarapacá, O’Higgins, and the Metropolitan Region (Santiago) because together they provide a geographically and demographically representative sample of northern, central, and southern Chile. These regions exhibit high and diverse concentrations of migrant populations, reflecting different patterns of settlement, socioeconomic integration, and access to public services. Tarapacá, as a border region in the north, concentrates one of the highest proportions of migrants in the country—approximately 23% of its population is foreign-born (38). O’Higgins represents a semi-rural area in central Chile where migration has increased significantly in the agricultural and service sectors, reflecting expanding settlement beyond major metropolitan centers (39). Meanwhile, the Metropolitan Region of Santiago hosts the largest and most heterogeneous urban migrant population, concentrating nearly 58% of all foreign residents in Chile (40). This territorial diversity enables the comparative analysis of different migration contexts and the identification of regional inequalities in food security, access to school feeding programs, and nutritional status among children and adolescents.

##### 2.1.1.1 Quantitative component

The main objective of this component was to characterize the conditions of food security, access to school feeding programs, and nutritional status of children and adolescents (C&A), comparing migrant and local populations, and examining territorial inequalities across the regions of Tarapacá, O’Higgins, and the Metropolitan Region.

###### 2.1.1.1.1 Materials and data sources

The quantitative component was developed through secondary analysis of national databases that provide information on the nutritional and food security situation of migrant children in Chile. Three main sources were integrated:

- National Socioeconomic Characterization Survey (CASEN 2022): CASEN provides information on households with children and adolescents under 19 years of age, including variables on poverty, food insecurity, and access to school feeding programs. For this study, adults were excluded, and the analytical subsample was restricted to children and adolescents under 19 years living in Chilean or migrant households. This subsample comprised *N* = 43,722 unweighted observations, representing 4,451,114 individuals after applying the official expansion factors.
- Monthly Statistical Records (REM, 2019–2021) REM contains data from Primary Health Care (APS) services, including nutritional risk assessments and diagnoses related to childhood malnutrition. This database was obtained through a transparency request to the Chilean Ministry of Health, as these data are not publicly available. For each year (2019–2021), we extracted all records for children and adolescents under 19 years of age and classified them by age group and nationality (Chilean vs. migrant). The total number of Chilean and migrant children and adolescents included in REM by age group and year is summarized in Table 1.

**Table 1:** Number of Chilean and migrant children and adolescents (<19 years) in REM, by age group and year.

Year	Age group (years)	Chilean, *n*	Migrant, *n*	Total, *N*
2019	0–5	731,232	14,790	746,022
	5–9	387,068	7,679	394,747
	10–19	250,358	9,205	259,563
	Total 2019	1,368,658	31,674	1,400,332
2020	0–5	610,054	13,065	623,119
	5–9	330,997	6,641	337,638
	10–19	189,553	7,902	197,455
	Total 2020	1,130,604	27,608	1,158,212
2021	0–5	538,873	9,369	548,242
	5–9	242,996	6,634	249,630
	10–19	163,611	7,556	171,167
	Total 2021	945,480	23,559	969,039

Own elaboration based on REM 2019, 2020, and 2021 (Ministerio de Salud de Chile). Subpopulation: children and adolescents under 19 years registered in the National Child Health Program and in the Comprehensive Adolescent Health Program, both categorized according to nutritional status.

###### 2.1.1.1.2 Construction of the food insecurity variable

The variable for food insecurity (FI) was constructed using the ELCSA module Latin American and Caribbean Food Security Scale (41), included in CASEN 2022 (42). The analytical sample for this variable comprised 20,885 observations with valid ELCSA information. Food Insecurity was classified into three levels: (a) Mild: Concern about food or reduction in food quality without significant reduction in quantity; (b) Moderate: Partial reduction in the quantity or diversity of foods available in the household; (c) Severe: Drastic reduction in food quantity, omission of meals, or entire days without eating. Households with migrant and Chilean children were categorized according to these levels of food insecurity. Proportions were estimated and cross-analyzed with multidimensional poverty indicators, region, and access to school feeding programs. Poverty indicators were grouped into three categories, extreme poverty, non-extreme poverty, and non-poverty, following the official criteria of the Ministry of Social Development and Family (43).

###### 2.1.1.1.3 Quantitative data analysis

Data processing was carried out using Stata 17 (44). For CASEN 2022, all analyses accounted for the complex survey design by applying sampling weights and stratification [svyset cod_upm pw = expr, strata(estrato)] to ensure representative estimates (43). The analyses included: (1) Description of the nutritional situation by nationality and region; (2) cross-tabulation of food insecurity (ELCSA) and poverty condition; (3) estimation of coverage in school feeding programs (PAE, JUNAEB); (4) territorial analysis of gaps between migrant and Chilean households; (5) integration of REM data to complement findings on nutritional morbidity and gastrointestinal conditions related to living standards and access to food; and (6) a statistical association between nationality and levels of food insecurity was evaluated using the weighted chi-square (χ^2^) test (fw). This analysis was applied exclusively to the food insecurity variable, as it was the only one designed to assess differences between population groups (Chilean households vs. migrant households).

##### 2.1.1.2 Qualitative component

The qualitative phase aimed to explore how migrant adults from Latin America and the Caribbean (LAC) living in Chile, along with healthcare providers (including migrant caregivers of children and adolescents and primary healthcare professionals working with migrant populations), experience access to primary healthcare services. Data collection took place between January and June 2023 across three Chilean regions with significant migrant populations: Tarapacá (north), the Metropolitan Region (center), and O’Higgins (south). Using a convenience sampling strategy, 42 semi-structured interviews were conducted following specific inclusion criteria for each participant group. Sample size was determined based on information saturation and feasibility, acknowledging that both migrants and healthcare workers often have limited availability to participate in research. Additional methodological details are presented in Table 2. As this was an exploratory qualitative component, representativeness was not the objective. Previous publications (45) have focused on adolescent sexual and reproductive health; in this study, we concentrate on findings related to the nutritional health of migrant children from the perspectives of their primary caregivers and healthcare teams. Due to time and ethical constraints, data were not collected directly from children.

**Table 2:** Participant’s characteristics by region (*N* = 42).

Group	Characteristic	Metropolitan Region	O’Higgins Region	Tarapacá Region
Migrant adults	Nationality	5 Venezuelan 2 Argentinian 1 Haitian	7 Venezuelan 1 Colombian	5 Venezuelan 2 Bolivian 1 Peruvian
	Gender	7 women, 1 man	8 women	8 women
	Average age	32 years	36 years	30 years
	Total participants	*n* = 8	*n* = 8	*n* = 8
	Total (Migrant adults)	N = 24		
Healthcare teams	Profession	Social workers 1 Nutritionist 1 Midwife 1 Nurse	4 Social workers 2 Cultural mediators 1 Nurse	4 Social workers 1 Nurse 1 Nutritionist
	Gender	6 women	3 women, 3 men	4 women, 2 men
	Total participants	*n* = 6	*n* = 6	*n* = 6
	Total (Healthcare teams)	*N* = 18		
	Total Participants	*N* = 42		

###### 2.1.1.2.1 Materials and participants

The study included a convenience sample of 42 in-depth interviews conducted with healthcare professionals (*n* = 18) and parents or guardians of migrant children and adolescents (*n* = 24). The inclusion criteria for migrant adults required participants to be of Latin American or Caribbean origin, of any gender, residents in Chile for less than ten years, and primary caregivers of at least one children or adolescent under 19 years old. The group of healthcare professionals comprised individuals providing direct care to migrant children and adolescents within primary healthcare settings, with at least one year of experience in their current role. Participant recruitment was carried out in collaboration with a non-governmental organization (NGO) specializing in migration issues, which facilitated access to eligible families and professionals. As a result, the sample included adults from Venezuela, Bolivia, Peru, Haiti, Argentina, and Colombia, reflecting the most common countries of origin among international migrants residing in Chile.

###### 2.1.1.2.2 Recruitment and data collection

All interviews were conducted in Spanish by the research team (AC and CR), lasted between 50 min and two hours, and were audio-recorded and fully transcribed after obtaining informed consent from participants. Interviews with healthcare teams were conducted online through Zoom, while those with adult migrants took place face to face in public spaces, participants’ homes or workplaces, or in facilities provided by collaborating non-governmental organizations, ensuring privacy and confidentiality throughout the process. The interview guide was developed by AC. Adult migrants were recruited through non-governmental organizations, and healthcare teams were invited to participate through healthcare service authorities that collaborated with the study.

###### 2.1.1.2.3 Qualitative data analysis

Once the interviews were transcribed verbatim and cross-checked against the original recordings, a thematic analysis was conducted. This approach enabled the interpretation of content based on themes, categories, and indicators previously defined by the research team, drawing on the study’s specific objectives and the guiding questions used in the research instruments. Atlas.ti 22 software (46) supported the coding and organization of the data. Coding was performed using both pre-established categories—derived from the research objectives and the main topics of the interview guides—and new codes that emerged inductively from the data. The entire research team reviewed the coding process, discussed discrepancies, and reached consensus on the final coding framework. All coding decisions were approved collectively. Quotations were translated into English by a native speaker, and all authors verified the translations to ensure fidelity to the original meaning. Following separate analyses of the quantitative and qualitative components, findings addressing common themes were integrated during the interpretation phase to provide a more comprehensive understanding of the research problem.

The study was approved by the Ethics Committee of Universidad del Desarrollo (Code #2022–92) and adhered to the ethical principles governing human subject research. All interviews were treated confidentially, and data were anonymized during transcription (i.e., using codes). Participants were informed about the study’s aims and procedures and signed an informed consent form confirming the voluntary nature of their participation. The research protocol included referral procedures for cases involving general or mental health needs, migration counseling, or potential identification of violence, abuse of minors, or human trafficking. None of the interviews required activation of this protocol.

## 3 Results

The findings combine both components (i.e., quantitative and qualitative) to increase the understanding of the nutritional status of migrant children and adolescents, and to comprehensively identify both the limiting and facilitating factors that influence the effective exercise of their right to access food and health nutrition. The results are organized by three dimensions: (1) nutritional status, food insecurity and its association with conditions of origin and displacement; (2) Food and Nutritional Insecurity (FNI) and barriers accessing healthy food in the context of arrival; and (3) lack of cultural acceptability and relevance in child nutrition programs that emerged mainly from the in-depth interviews, for this reason, the dimension is mainly supported by qualitative data.

### 3.1 Nutritional status, food insecurity and its association with conditions of origin and displacement

According to CASEN 2022, migrant children and adolescents (NNA) exhibit a more favorable nutritional profile than their Chilean peers: over 91% fall within the normal range, compared with 82% among Chilean NNA. However, undernutrition or risk thereof (2.9%) is twice as prevalent among migrants as in the national population (1.4%), reflecting economic vulnerability and barriers to accessing adequate and quality food (See Table 3).

**Table 3:** Nutritional status of children and adolescents (NNA) by nationality (CASEN 2022).

Category	Chilean NNA (%)	Migrant NNA (%)
Underweight or at risk	1.4	**2.9**
Normal	82.3	**91.4**
Overweight	**14.0**	4.3
Obese	**1.9**	0.6
Does not know	0.4	0.9

Own elaboration based on CASEN 2022. Subpopulation: children and adolescents (<19 years). Complex sampling design adjusted for expansion. Bold values indicate higher prevalence or coverage between groups.

**Figure 1:**
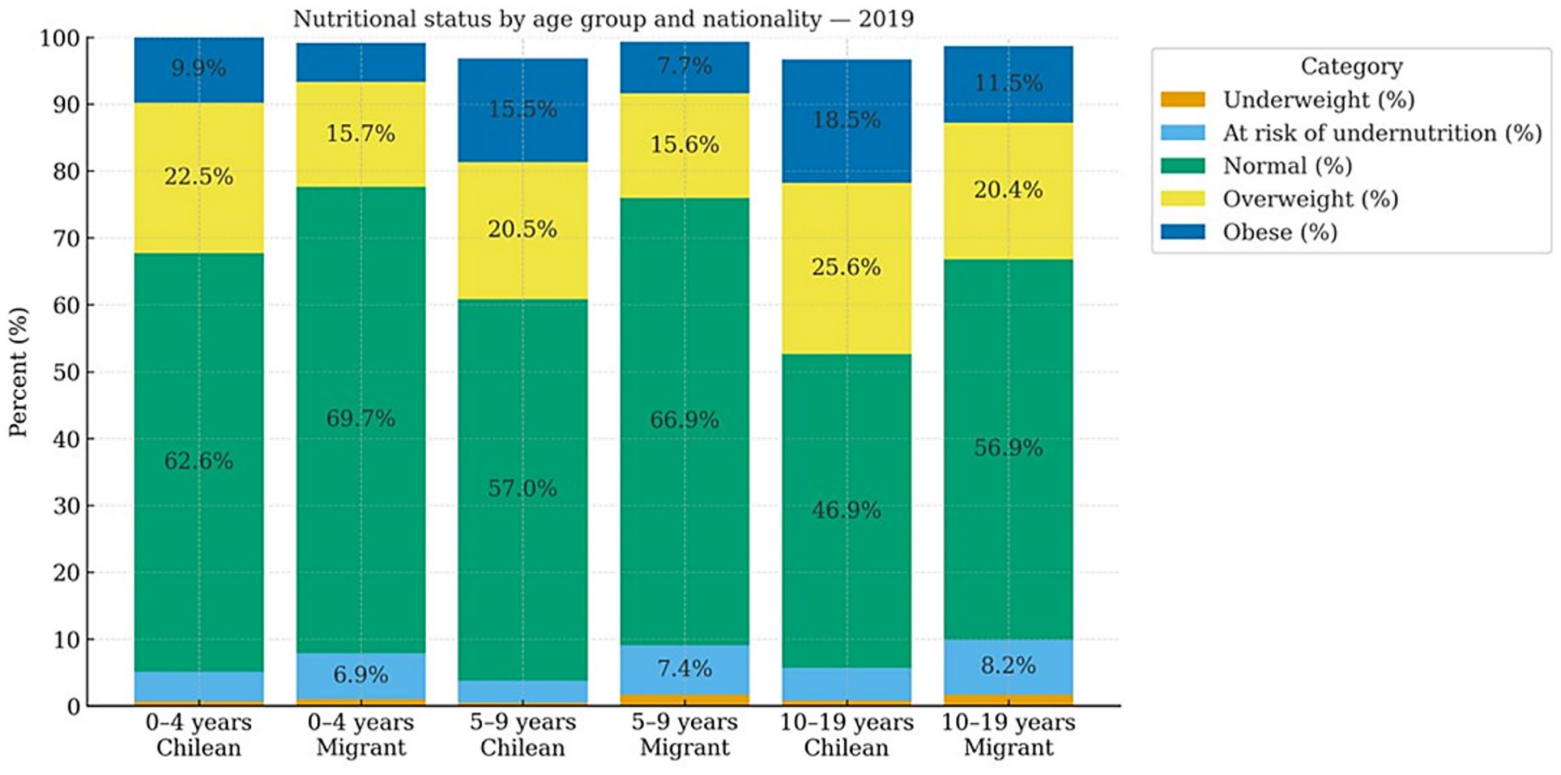
Nutritional status by age group and nationality (2019). Own elaboration based on REM 2019. Ministry of Health, Chile.

**Figure 2:**
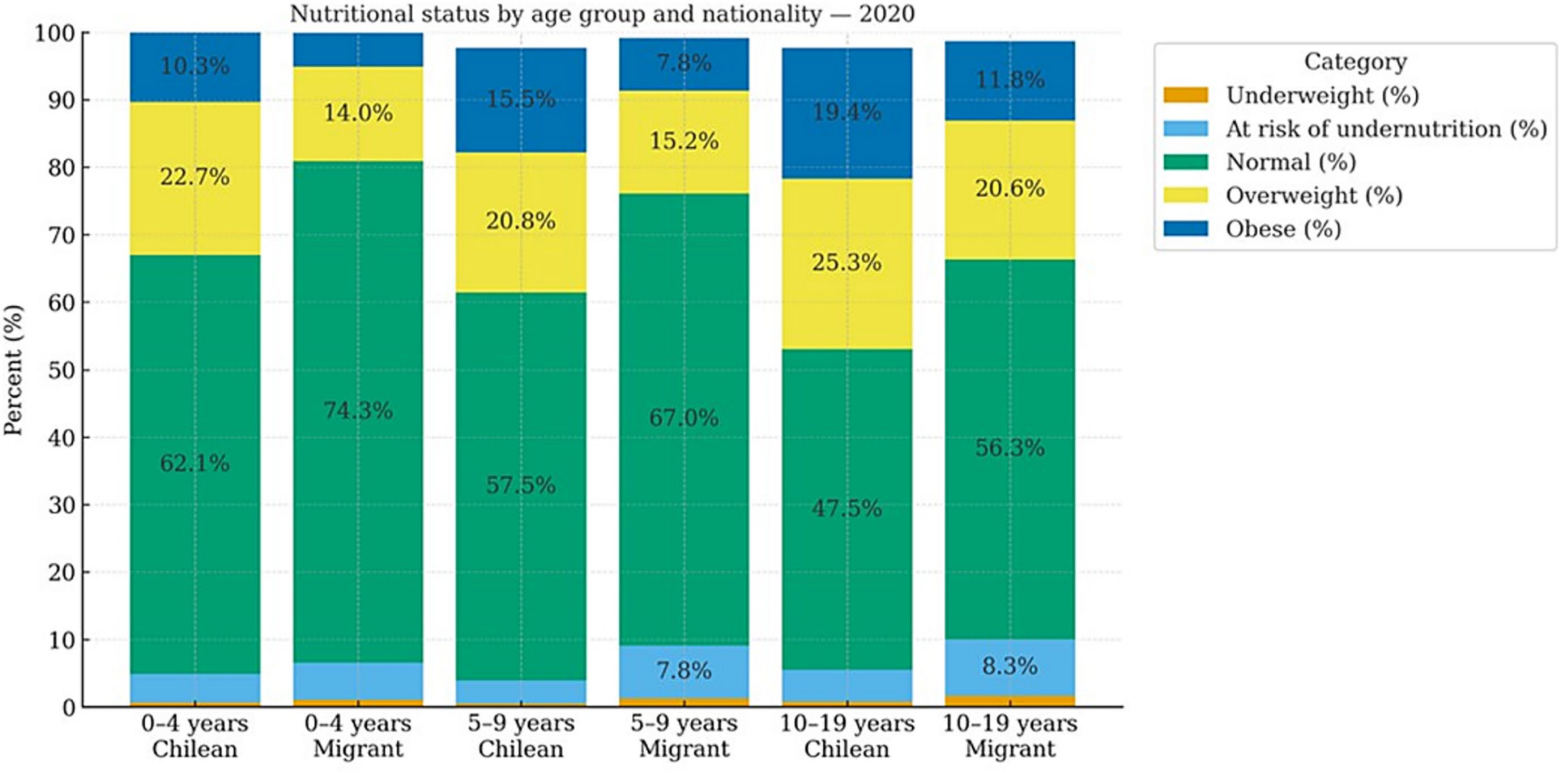
Nutritional status by age group and nationality (2020). Own elaboration based on REM 2019. Ministry of Health, Chile.

**Figure 3:**
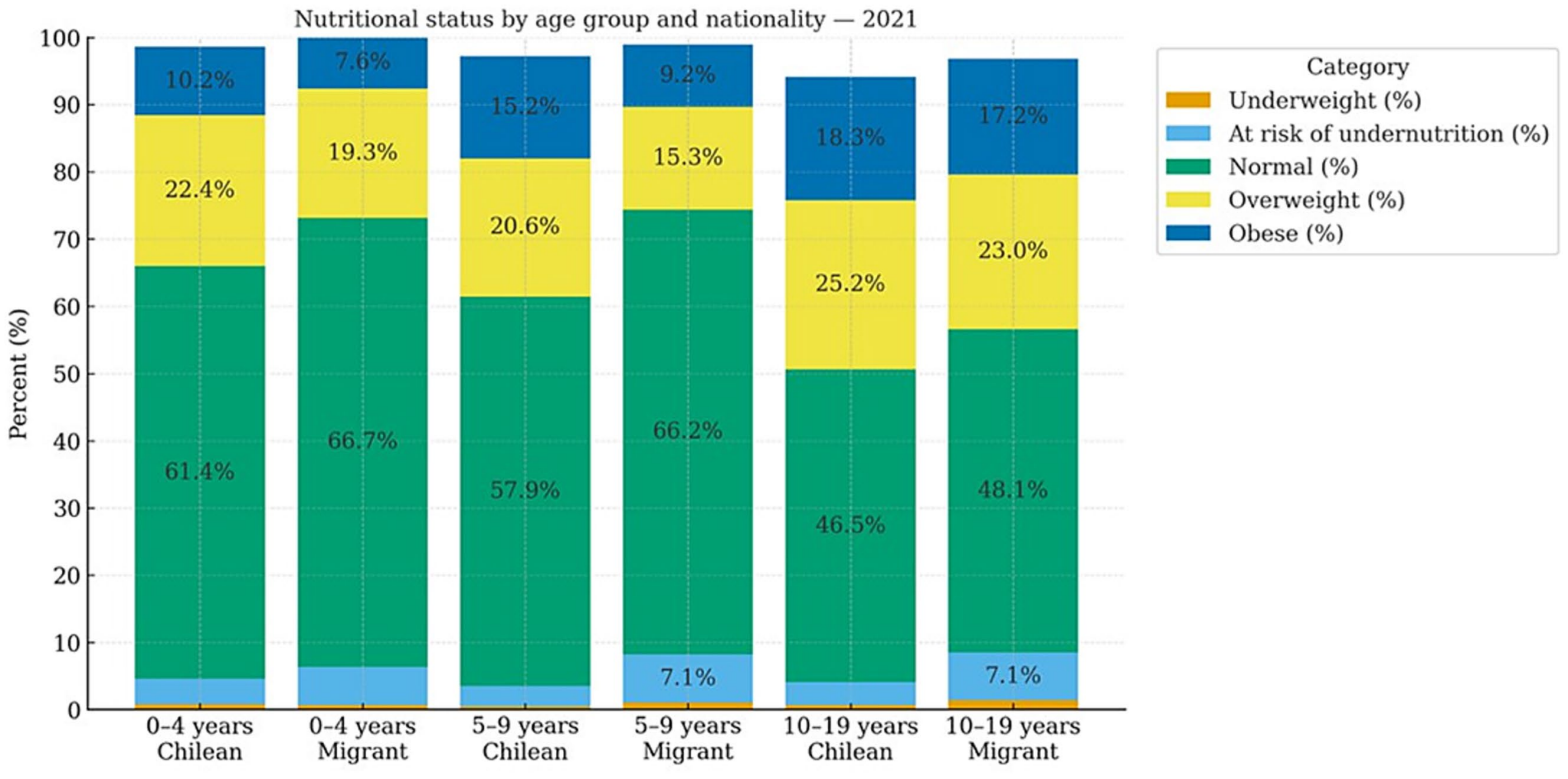
Nutritional status by age group and nationality (2021). Own elaboration based on REM 2019, Ministry of Health, Chile.

This trend is confirmed when analyzing the Monthly Statistical Records (REM, 2019–2021), which reveal consistent differences by nationality and age (See Figures 1–3).

In 2019, a clear pattern emerged across childhood and adolescence (ages 0–4, 5–9, and 10–19): undernutrition was concentrated among the migrant population, which exhibited consistently higher rates of both undernutrition and malnutrition compared to Chilean peers (ages 0–4: 6.9 and 1.0% vs. 4.4 and 0.7%; ages 5–9: 7.4 and 1.7% vs. 3.3 and 0.5%; ages 10–19: 8.2 and 1.7% vs. 4.9 and 0.8%). Conversely, overnutrition was more prevalent among Chilean children and adolescents, who showed higher levels of overweight and obesity across all age groups (See Figure 1).

In 2020, the same pattern persisted across childhood and adolescence: deficit malnutrition remained concentrated among the migrant population, with higher proportions of both risk of undernutrition and undernutrition in all age groups (0–4 years: 5.5 and 1.1% vs. 4.2 and 0.7% among Chileans; 5–9 years: 7.8 and 1.3% vs. 3.4 and 0.5%; 10–19 years: 8.3 and 1.7% vs. 4.7 and 0.8%). In contrast, excess malnutrition continued to be more prevalent among the Chilean population, with higher levels of overweight and obesity across all age groups, as shown in Figure 2.

In 2021, the same pattern persisted: deficit malnutrition remained concentrated among the migrant population. The risk of undernutrition was higher among migrants across all ages (0–4 years: 5.7% vs. 3.8% in Chileans; 5–9 years: 7.1% vs. 3.0%; 10–19 years: 7.1% vs. 3.4%). Undernutrition was also higher in migrants aged 5–9 years (1.1% vs. 0.5%) and 10–19 years (1.4% vs. 0.7%), while rates among those aged 0–4 years were similar (0.7% vs. 0.8%). Nonetheless, the overall deficit (risk plus undernutrition) remained greater in migrants due to the higher risk component. In contrast, excess malnutrition (overweight and obesity) continued to be higher among Chileans in all age groups, though the gap narrowed among adolescents, as shown in Figure 3.

To understand what could be explaining the difference between children’s migrant nutritional health and Chilean children we analyzed the content of in-depth interviews conducted to adult migrants and health care teams. Our qualitative findings revealed a shared understanding among health teams and migrant populations that living conditions, both in the country of origin and during migration through unauthorized routes, adversely affect the nutritional health of families, particularly children and pregnant women. Malnutrition, especially among Venezuelan migrants, is closely linked to prolonged periods of limited access to food, as reflected in the testimonies of the following health professionals which may be extend once they arrived in the destination country:

*“Some children have arrived in Chile with malnutrition; they have arrived with certain illnesses that have worsened along the journey. The trip is extremely long, and therefore they come in precarious conditions. Pregnant mothers, with small babies, without health checkups, and underweight.” — (Tarapacá Region, nurse).*

Similarly, another health professional emphasizes how food insecurity during the journey contributes to poor nutritional outcomes, particularly among children:

“I think that due to the journey, an important issue is malnutrition. Many tell me that doctors have said their child is malnourished, and that’s obviously because — what can you eat there? There isn’t a balanced diet — no vegetables, no fruits — it’s probably mostly flour-based foods. So yes, malnutrition is an important issue.” — (Metropolitan Region, social worker)

Another participant highlighted the extreme physical risks faced by migrants along the route, particularly for pregnant women and infants, underscoring the intersection of violence, hunger, and health vulnerability:

*“The real risk they face crossing all that desert, the altitude — one (user) once told me that the risk of dying was the same: ‘either I die walking, or I die of hunger in Venezuela.’ So of course, her story makes sense. […] We receive patients who arrive at 35 weeks of pregnancy — basically ready for the picture (for childbirth) — and who have just crossed. Or young women who give birth at the border.” — (Tarapacá Region, social worker)*

Furthermore, migrant families acknowledge that food was one of the main concerns they faced during the migratory journey, and, in some cases, they mentioned it as one of the reasons that led them to decide to migrate, even thru unauthorized crossings. As one participant recounts, maintaining an adequate diet during the journey was extremely difficult due to limited access to nutritious food and the need to rely on what was available along the way:

“*Well, food was always an issue. When I left Venezuela, I brought my little breads, my arepas, but along the way, whenever I reached a place and saw bread, I would buy some — bread, cookies — everything was light food, like a bit of cheese, water, juice, all like that until I could finally get here and eat properly… but yes, I spent a long time eating like that.” — (Tarapacá Region, migrant women)*

A migrant who moved from Venezuela, emphasized that the persistent food shortages and general deterioration of living conditions in Venezuela were key motivations for deciding to leave:

*“…And with all the problems Venezuela has had with food, health, all of that — we went through all of it and saw everything, and that’s why we left. Seeing that there was no food was terrible.” — (Metropolitan Region, migrant women)*

Others highlighted the extreme conditions endured during the journey, where hunger and exposure to harsh climates compounded the physical and emotional toll on families, especially children:

*“But yes, we suffered from — well, at that time we suffered from hunger and extreme cold. Even the children, when we arrived in Bolivia, it was so cold they started to cry. The same happened in Colchane, because the cold there is terrible — and with hunger, with nothing to give them to warm their bodies.” — (Tarapacá Region, migrant women)*

Parent’s possibility of accessing food such as meat and chicken, for themselves and their children, was mentioned by some participants as one of the reasons that sustained the migration project, even when it involved walking through the desert:

*“And he (her son) now sees that at least in another country there is (food), yes… we have to walk, because we had to walk a lot, and here if he wants to have a soda, he knows he can have one and eat well. In Venezuela, chicken was for rich people, but here you can eat chicken, meat. It takes effort, but it’s possible.” — (O’Higgins Region, migrant women)*

As this section shows, quantitative and qualitative data converge on the existence of unmet food needs in the context of origin and migration experienced by children and adolescents in LAC. Access to food is also highlighted as a reason for migration among families migrating through unauthorized border crossings with children.

### 3.2 Food and nutritional insecurity (FNI) and barriers to accessing healthy food in the context of arrival

At the national level, the CASEN 2022 results reveal a marked disparity in food security between migrant and Chilean households. Severe food insecurity affects 28.1% of migrant households—almost twice the rate observed among Chileans (15.9%)—while mild food insecurity is more prevalent among Chilean households (58.3% versus 45.1%). These differences are statistically significant according to Pearson’s chi-squared test [χ^2^(2) = 169.3, *p* < 0.001], confirming the greater vulnerability of the migrant population to structural food deprivation (see Table 4).

**Table 4:** Food and nutritional insecurity (FNI) in households with children and adolescents (CASEN 2022).

ELCSA Category	Chileans (%)	Migrants (%)
Mild	58.3	45.1
Moderate	25.8	26.8
Severe	15.9	**28.1**

Own elaboration based on CASEN 2022. Subpopulation: children and adolescents (<19 years). Complex sampling design adjusted for expansion. χ^2^ = 169.3, *p* < 0.001. Bold values indicate statistically significant differences based on Pearson’s chi-square test.

The relationship between poverty and child food insecurity demonstrates a clear socioeconomic gradient among both Chilean and migrant children and adolescents. As households transition from conditions of extreme poverty to non-poverty, the prevalence of severe food insecurity declines, while cases of mild food insecurity become more common, indicating a shift in intensity rather than a complete resolution of vulnerability. Nevertheless, across all poverty strata, migrant children consistently exhibit a more adverse food insecurity profile compared to their Chilean counterparts, underscoring the compounded effects of socioeconomic disadvantage and migration status (See Table 5).

**Table 5:** Relationship between poverty and child food insecurity (CASEN 2022).

Poverty status	Mild FI (%)	Moderate FI (%)	Severe FI (%)	Total (%)
Chilean children and adolescents				
Extreme poverty	**38.0**	**28.9**	33.1	100
Non-extreme poverty	**44.0**	28.2	27.8	100
Non-poor	**61.0**	25.4	13.6	100
Migrant children and adolescents				
Extreme poverty	29.1	22.1	**48.8**	100
Non-extreme poverty	34.5	**29.1**	**36.4**	100
Non-poor	49.1	**26.9**	**24.0**	100

Own elaboration based on CASEN 2022. Subpopulation: children and adolescents (<19 years). Bold values indicate higher prevalence or coverage between groups.

In extreme poverty, almost half of migrant children experience severe food insecurity (48.8%), compared to 33.1% among Chileans (+15.7 percentage points). Among those in non-extreme poverty, severe food insecurity affects 36.4% of migrants versus 27.8% of Chileans (+8.6 points). Even in the non-poor group, severe food insecurity among migrants remains high at 24.0%, exceeding the 13.6% observed in Chileans (+10.4 points). This pattern suggests a shift in the distribution toward higher levels of severity for migrant children, even as poverty conditions improve. Moderate food insecurity shows more minor differences between groups (e.g., non-poor: 26.9% among migrants vs. 25.4% among Chileans). In comparison, mild food insecurity is consistently lower among migrants (non-poor: 49.1% vs. 61.0%), consistent with a greater relative concentration in the moderate/severe categories. Taken together, the data reveal a consistent pattern: as poverty conditions improve, the severity of food insecurity declines. However, the distribution among migrant children remains disproportionately concentrated in the moderate and severe categories. This relative disadvantage persists across all poverty levels, including extreme poverty, non-extreme poverty, and non-poor households, reflecting a sustained disparity in the severity of food insecurity between migrant and Chilean children despite overall socioeconomic improvements (See Table 5).

When analyzed the access to Chilean School Feeding Programs (provided by JUNAEB) we found that access to free breakfast and lunch is higher among migrant children and adolescents (≈63–66%) than among Chileans (≈51%), reflecting concentration in vulnerable schools with a high presence of migrant students. Regarding access to school meals, it is noteworthy that the concentration of food insecurity (FNI) among migrant children appears even though the use of breakfast, lunch, and snack benefits is higher among migrant children than among Chilean children (See Table 6).

**Table 6:** Access to school feeding programs (PAE – JUNAEB) (CASEN 2022).

Food program (2022)	Chileans (%)	Migrants (%)
Free breakfast	50.7	**62.9**
Free lunch	51.8	**65.9**
Free afternoon snack (“once”)	11.3	**17.6**
Free school snack	23.1	**30.2**

Own elaboration based on CASEN 2022. Subpopulation: children and adolescents (<19 years). Bold values indicate higher prevalence or coverage between groups.

School feeding coverage for migrant children and adolescents shows a pattern of territorial targeting that responds to specific contexts of vulnerability but still leaves gaps in daily nutritional continuity. In Tarapacá, more than 70% receive free breakfast or lunch and 47% receive school snacks, reflecting a strong response in a border region with a high concentration of migrant families. In O’Higgins, coverage is also high (≈74%), driven by rurality and the presence of migrant agricultural households where schools serve as the main anchor for food security. In the Metropolitan Region, although levels are lower, migrants still maintain a relative advantage due to targeting in more vulnerable municipalities with a high concentration of foreign population. However, this scheme primarily prioritizes two meals (breakfast and lunch) and leaves the afternoon snack and dinner uncovered, meaning that caloric and micronutrient protection is not sustained throughout the day, particularly in households experiencing food insecurity (See Table 7).

**Table 7:** School feeding access by region and nationality CASEN 2022.

Region/Program	Nationality	Free breakfast (%)	Free lunch (%)	Free afternoon snack (“once”) (%)	Free school snack (%)
Tarapacá	Chileans	53.2	53.3	10.3	30.5
	Migrants	72.2	74.4	16.9	47.3
O’Higgins	Chileans	54.0	55.2	11.2	21.9
	Migrants	73.7	73.5	26.3	34.5
Metropolitan Region	Chileans	41.0	42.7	9.6	19.3
	Migrants	58.3	61.3	16.6	24.4

Own elaboration based on CASEN 2022. Subpopulation: children and adolescents (<19 years).

The second main finding, on which there is consensus among both families and health teams, refers to the living conditions faced by some migrant families upon arriving in Chile. These conditions refer to food insecurity and often prevent them from accessing healthy food, drinking water, and basic facilities for food preparation. As one health professional explained:

*“Sometimes pregnant women arrive underweight. For example, not long ago, a young woman from Peru came hoping to improve her quality of life, send money to her children back home, but she became pregnant in Chile. That dream of being able to contribute disappeared because once she got pregnant, her employer dismissed her. She had nowhere to live, nothing to eat, and we were left asking ourselves, what can we do? She’s underweight, and what can we do?” (Tarapacá Region, nurse)*

A key structural factor identified is the informality of migrant employment and the irregular legal status of many who have entered Chile in recent years. These conditions have been linked to the expansion of informal settlements known as *tomas*, where health teams frequently detect not only poor access to healthy food but also limited access to clean water and a high presence of parasitic and dermatological diseases associated with animal contact:

*“There are many gastrointestinal illnesses caused by ectoparasites and issues related to food handling. There are also ectoparasites because of the packs of stray dogs living in the settlements. We see a lot of skin diseases, not only among migrants but among everyone living there. Access to potable water is limited; municipal water trucks bring water, which people use mainly for cooking, while what remains is used for hygiene or cleaning. There are also many pets and garbage dumps, so the environment is very unsanitary.” (Tarapacá Region, social worker)*

These environmental conditions—presence of animals, waste accumulation, and limited access to sanitation and safe water—constitute major health risks for people living in informal settlements, many of whom are migrants:

*“Many migrants live in settlements. As you can imagine, these places lack basic services—no water, no electricity, nothing. When water is delivered, sometimes not everyone receives it, and that’s where the problems start. Water is essential for cooking, bathing, cleaning. People in these settlements are more exposed to diseases transmitted by vectors like mice, flies, lice, or nits, and all of that affects them.” (Tarapacá Region, social worker)*

However, it is important to note that not all migrant families live in informal settlements or face such precarious conditions. In fact, some families interviewed, even while living in housing with basic services and having stable jobs, still reported barriers to accessing adequate food due to economic constraints and time limitations related to food preparation and childcare. For example, one woman who lost her job during 2020–2021 became responsible for two girls, her younger sister and her daughter, and described the hardship of ensuring food security:

*“They paid me 5,000 pesos* (5USD)*, and I didn’t care if it was little; I just needed to clean houses so I could get money or something to eat. Sometimes people gave me food instead of money. I would much rather the girls ate. I didn’t eat much myself. Sometimes I’d cry at night thinking about what to do, and I even thought about going back to Bolivia.” (Tarapacá Region, migrant women)*

Time management and the incompatibility between work demands and caregiving responsibilities emerge as another critical barrier to ensuring healthy child nutrition, regardless of where they live. One participant, a mother of three working long hours in a restaurant, described having to leave her children alone all day, without support to prepare meals safely:

*“I work from ten in the morning until ten or eleven at night, and they’re alone all day. They tell me, ‘Mom, the food is cold, I don’t like it,’ and my little one became undernourished because her siblings don’t know how to take care of her. She’s only four years old. It’s very hard to leave her alone, but if I don’t work, they don’t eat.” (Tarapacá Region, migrant women)*

Other families echoed similar experiences, emphasizing the lack of support networks that forces them to leave children alone, relying on older siblings to reheat or serve food:

*“Yes, there are times when they’re alone. We always have to make sure the windows are closed because accidents have happened. We leave everything ready for them to eat.” (Metropolitan region, migrant men)*

Furthermore, both families and health teams reported some barriers to accessing school feeding programs provided by the Chilean state through the Junta Nacional de Auxilio Escolar y Becas (JUNAEB). Administrative barriers, particularly irregular migration status and the absence of a national identification number (i.e., RUT), prevent some children from being registered in social service systems and therefore from receiving school meals:

*“With JUNAEB support, they could get food, but the problem is that some schools require enrollment in the social registry. Families who entered through irregular border crossings don’t have a national ID or social registry. Whether a child receives food depends on the school’s discretion—if there are extra meals, they might get one, but it shouldn’t be that way.” (O’Higgins Region, social worker)*

For many families, school meals play a crucial role not only in meeting nutritional needs but also in supporting the social and cultural integration of migrant children:

*“At school, they get meals, though they’re still getting used to Chilean food—it’s very different. They try to mix foods, a Venezuelan dish at home and a Chilean one at school. I asked the school director to make sure they received meals since both parents work, and school lunches are essential.” (Metropolitan Region, migrant women)*

Through this experience, school feeding programs also serve as a medium for learning and cultural adaptation:

*“I like that they include him in the school cafeteria because one day he came home excited saying, ‘I ate boiled eggs!’ Now he asks for them for breakfast at home because he learned to eat them at school.” (Metropolitan Region, migrant women)*

In summary, barriers to accessing adequate nutrition for migrant families in Chile are multidimensional. They extend beyond economic limitations to include inadequate living conditions such as lack of potable water, sanitation, or cooking facilities, as well as time constraints linked to work and caregiving. Moreover, administrative barriers tied to migration status restrict access to public feeding programs, leaving some children without guaranteed access to sufficient and nutritious food.

### 3.3 Lack of cultural acceptability and relevance in child nutrition programs

Thirdly, both health teams and families recognize the existence of cultural differences between the dietary guidelines promoted by child health and school feeding programs and the food cultures present among migrant families. Meal schedules, types of preparations, and food combinations often differ, creating contradictions and tensions in the relationship between families and health professionals. As one health professional explained:

*“The main issue with food is the emphasis on making it healthy. Although it represents a major change from their home country, their traditions, and their usual foods, some people have found this adaptation difficult. For example, with Venezuelan families, here in Chile snacks usually consist of milk or juice, especially at school, while they bring arepas or a full meal instead. That change has been challenging for them because here children bring fruit or bread for mid-morning, whereas they bring arepas and cooked food, in addition to the lunch provided at school.” (Tarapaca Region, nutritionist)*

A nurse added that neither professionals nor the programs themselves often question the cultural adaptability of the nutritional guidelines given to families. These guidelines rarely account for the diversity of food cultures in areas with a high presence of migrant families. Referring to Haitian families, she explained:

*“When they’re told to make baby purées with squash, carrots, or artichokes, the Haitian immigrant doesn’t know what artichokes are. It’s hard to follow instructions that involve ingredients they’ve never seen before. As professionals, we should ask ourselves: if they don’t eat artichokes, how can they replace them? We don’t usually think about that. Sometimes patients tell me they don’t know how to prepare these foods. They say, ‘I give my child something else, he eats it well, and nothing bad happens.’ These situations can have long-term effects, so we need to think about how to address them, how to convince and include these families in the system.” (O’Higgins Region, nurse)*

Families also reported difficulties in understanding and following the dietary recommendations provided at health centers, especially concerning the introduction of solid foods for infants. There are contradictions between the advice received from health professionals and the practices followed in their countries of origin. One woman, who had a baby in Chile and an older child born in Colombia, shared her experience:

*“For example, they gave us a paper explaining what we should eat, but in my country, we have customs that say some foods are bad for babies. That’s why my baby only ate white soup. Where I’m from, beans are bad for babies, but here at the hospital they said it was fine to give beans. I couldn’t do it. I thought, how can I give him something that I know will harm him? So I never followed those instructions because I know they’re not right.” (O’Higgins Region, migrant women)*

This lack of cultural acceptability not only creates tension with health teams but also leads some families to abandon nutritional check-ups altogether, especially when attending health centers conflicts with their work schedules. As a Venezuelan father of a six-year-old girl explained:

*“The nurses sometimes tell me there are workshops for us to learn about child nutrition when they see kids who are too thin or overweight. They invite us to attend, but I work during the week, and so do they, and besides, I know they don’t understand the kind of food we eat, so I don’t go.” (Metropolitan Region, migrant men)*

Health professionals are aware of this reality and acknowledge that some nutritional consultations end up being performed simply to meet institutional requirements, without meaningful engagement from families:

*“Migrant women think, ‘If I already have children, why attend a workshop?’ They don’t see the point. We give them the same advice during appointments, but it’s mostly to feel that we’ve done our job, even if they don’t follow through. Then we schedule them for two months later.” (O’Higgins, social worker)*

Another health professional pointed out an often-overlooked dimension of food practices in the migration context—the emotional and cultural aspects of nutrition and the transmission of cultural identity through food. This dimension deeply affects migrant families, as illustrated in the following case:

*“We have to understand and adapt to cultural differences. When I provide a feeding plan, I don’t tell them to eat potatoes and rice like we do in Chile. They adapt, and we must be flexible, because food is one of the few things that keeps them connected to their culture. Continuing to cook what they used to eat, teaching their children about those foods, is very meaningful for them. For instance, I have a Chilean patient who had a child with a Haitian man. She decided from the very first meal to feed her baby Haitian food because, she said, it might be the only way for him to know his heritage. I think that’s important—we adapt, we compromise, but we also know that feeding practices can have long-term health effects, like allergies, so we need to find a balance.” (Metropolitan Region, social worker)*

Questioning and disregarding this emotional and cultural dimension of food practices can lead to the delegitimization of parenting styles among migrant families, reinforcing stereotypes and tensions between them and health professionals:

*“For migrant families, being questioned is very difficult because, just as their food is questioned, their parenting is questioned too—and we’ve seen that even among colleagues.” (Metropolitan Region, social worker)*

Finally, some health teams have begun developing small initiatives to adapt dietary guidelines to the realities of migrant families, even though these efforts remain isolated:

*“We’ve been exploring the idea of organizing gatherings where parents can share and taste different kinds of food. At the CESFAM, for instance, we thought about inviting a Haitian family to cook something during an activity, and Chilean families could prepare something too, so everyone can try, learn, and value these different foods.” (O’Higgins Region, cultural mediator)*

This emergent qualitative theme shows that migrant families in Chile face significant barriers to accessing healthy nutrition. These barriers stem from both pre-existing conditions related to their migration trajectories, particularly for those who migrated through irregular routes—and from challenges encountered after arrival, such as economic limitations, housing conditions, work schedules, and lack of time to prepare and provide food for children. Administrative barriers also exclude migrant children from the national school feeding program, a key mechanism in Chile for reducing malnutrition. Finally, the limited cultural acceptability of nutritional programs and the lack of recognition of diverse food cultures contribute to misunderstandings and reinforce inequities. Addressing these challenges requires integrating an equity-based and intercultural perspective into child nutrition policies and programs to mitigate the long-term impacts identified in this study.

## 4 Discussion

This study reveals the coexistence of multiple forms of malnutrition among children and adolescents in Chile illustrating a disparity in nutrition realities faced by migrant children and adolescents. Firstly, the findings show that qualitative and quantitative data are consistent about the existence of food insecurity and undernutrition that affect these groups in context of origin and displacement. From quantitative data migrant children from LAC present a higher prevalence of undernutrition and risk of undernutrition compared with Chilean children, despite having lower rates of overweight and obesity. Qualitative data shows that lack of food can be considered a pull factor to migrate from countries that are living a nutritional crisis as Venezuela (47). These results are consistent with regional evidence from Peru and Colombia, where newly arrived Venezuelan children show elevated levels of wasting and anemia (10, 18). Worryingly undernutrition not only affects children and adolescent, but also pregnant women, condition that can lead maternal complications and long-term health issues for the child (16, 48). Despite evidence of the importance of solidarity networks for coping with food insecurity during transit (49), access to food for children is also not guaranteed during displacement, especially for people migrating through non-authorised crosspoints.

As the study evidence national programs are focused on addressing overweight and obesity (50) among children and adolescents, overlooking a vulnerable subgroup whose right to adequate nutrition remains poorly understood and unmet (51). In fact, Chile has achieved significant progress in controlling undernutrition over past decades through programs such as the National Complementary Feeding Program (PNI) and the school feeding program managed by the Junta Nacional de Auxilio Escolar y Becas (JUNAEB) (34, 35). These interventions have contributed to a steady reduction in childhood stunting and underweight since the 1990s (51, 52). However, the country now faces one of the highest childhood obesity rates globally, with over half of preschool children classified as overweight or obese (53). Consequently, public policies have shifted almost exclusively toward combating overnutrition, leaving little institutional attention to the resurgence of nutritional deficits in specific subpopulations, including migrant children (54).

Secondly, the study shows that food and nutritional insecurity does not disappears in the host country. In Chile, the phenomenon appears to be reinforced by economic vulnerability, spatial segregation and administrative barriers, including migration status, that limit access to child health and school feeding programs (7). The CASEN 2022 data reveal that nearly one in three migrant households with children experiences severe food insecurity, a rate nearly double that of Chilean households. This suggests that migrant families, even those not classified as poor by income, remain disproportionately exposed to food deprivation and instability in comparison to Chilean families (7). Previous evidence (4) had shown the inequalities faced by migrant children in terms of multidimensional poverty and access to health services. The data reported here are consistent with these findings and add the factor of food insecurity, revealing new forms of inequality. Even though poverty, spatial segregation and the lack of basic conditions such as access to drinking water and sanitation affect the food security of children, regardless of whether or not they are migrants, the data show that these factors override administrative barriers such as migration status and the lack of support networks, which are key to ensuring safe and quality food. Otherwise, data reported shows that these disparities are not only economic but also institutional. Our study identified that many migrant families, particularly those in irregular migration status, cannot access public food programs such as JUNAEB due to the lack of a national identification number (RUT) or enrollment in the social registry. Similar barriers have been reported in other Latin American contexts where migrants are excluded from social protection systems (2, 19). The achievements made in controlling malnutrition through school feeding programmes (35) must be extended to emerging vulnerable groups such as migrant children, especially those from countries without food security (55) or those who are in an irregular migratory situation (56). Lack of regular status has been reported as a risk factor not only for pregnancy among migrants, but for different pathologies, especially contexts in which access to healthcare requires significant out-of-pocket expenses. A study comparing the economic outcomes of Latin American migrants in Spain and the United States showed that legal status has a more pronounced effect on undocumented migrants than on documented migrants, especially in the United States (57). In this case, the data show that when immigration status is a barrier to accessing school feeding programmes, it can exacerbate the effects of food insecurity in migrant children.

Thirdly, beyond institutional exclusion, qualitative data highlight that health and nutrition programs in Chile often lack cultural relevance and does not consider the food practices and knowledge of migrant communities. Migrant families reported difficulties in understanding or accepting the dietary guidelines provided by primary health centers, which are designed from a Chilean perspective and do not incorporate the cultural diversity of migrant populations. This echoes findings from Hun et al. (5), who emphasize that dietary acculturation processes can generate tension between health recommendations and traditional food practices (58, 59). Health professionals in this study acknowledged that national programs rarely question the cultural adaptability of their recommendations or offer alternatives that respect diverse food habits, particularly important for the migrant communities. Despite these difficulties, school feeding programmes are viewed positively, especially by families who do not have sufficient support networks to ensure safe spaces for feeding or who are forced to prioritise work over childcare. Efforts to make health service dietary guidelines more flexible and learn about the food traditions of migrant communities are viewed positively by migrant families and health teams.

The intersection of migration, social inequality, and cultural dissonance produces a “double nutritional vulnerability” for migrant children. On one hand, they face a risk of undernutrition due to material deprivation, food insecurity, and barriers to public programs. On the other hand, prolonged exposure to Chile’s food environment may eventually shift them toward overweight and obesity, replicating patterns observed in other migrant populations over time (24). This transition reflects a form of “nutritional acculturation,” in which traditional dietary patterns are progressively replaced by energy-dense, processed foods that are more affordable but nutritionally poor (29, 30).

Chile’s public health response thus faces a paradox. While the country continues to strengthen obesity prevention (60), it lacks a complementary approach that ensures early detection and treatment of undernutrition among migrant children. This omission risks deepening social and health inequalities. As noted by the FAO, PAHO, and WFP (61), the persistence of undernutrition among migrant and displaced children across Latin America signals the need for host countries to adopt a dual-focus strategy that addresses both ends of the malnutrition spectrum.

To close these gaps, Chile must advance toward an intercultural and equity-based model of nutritional health. Guaranteeing access to school feeding programmes for children, regardless of their immigration status, and prioritising their enrolment in social protection and primary care services are key actions to reverse the effects of food insecurity that they may have faced in their countries of origin and continue to face in the host country. This includes expanding access to JUNAEB; adapting nutritional guidelines to recognize diverse food cultures; and training health professionals in community-based interventions. Evidence from this type of interventions in Latin America suggests that integrating cultural identity into nutrition education can enhance adherence and improve child feeding practices (62, 63). Similarly, intercultural adaptations to school feeding programs, such as the inclusion of migrant families in menu design and preparation, have shown promise in promoting inclusion and dietary diversity (9).

This study has several significant limitations. First, because we relied on secondary data collected by the Chilean government, our analyses were constrained by the structure and quality of those existing sources. While the CASEN survey is publicly accessible, the REM dataset required a special access request. As a result, the availability of specific variables and data elements may be limited for researchers seeking to replicate or extend this work. Such access restrictions may also impact the comparability of future analyses. Despite these limitations, this work provides the first mixed-methods evidence on differences in the nutritional status of migrant and Chilean children and adolescents, addressing a critical gap in the literature. The qualitative component has a few limitations. We recognize potential bias in including only three regions in Chile (i.e., Tarapacá, the Metropolitan Region, and O’Higgins), as this may not reflect what happens across the entire country. Furthermore, we did not conduct in-depth interviews with children or adolescents; we only interviewed caregivers and professionals. Thus, we may have lost some essential details about migrants’ nutritional health. Lastly, due to the nature of qualitative methods, we cannot generalize these findings, as we used convenience sampling to identify nuances and subjectivity rather than generalization or patterns.

In summary, Chile’s current focus on childhood overweight and obesity, while justified by epidemiological trends, must be complemented by policies that address the silent and growing problem of undernutrition among migrant children. This requires moving beyond a one-size-fits-all model to a rights-based approach that ensures equitable access to adequate food and health care for all children living in Chile, regardless of their nationality or migration status. Addressing undernutrition today is essential to prevent the reproduction of health inequalities and to uphold Chile’s long-standing commitment to social protection and child well-being.

## Data Availability

The datasets presented in this article are not readily available due to ethical and confidentiality reasons. Requests to access the datasets should be directed to the corresponding author, Alejandra Carreño-Calderón, a.carreno@udd.cl.
